# Cuproptosis-Related LncRNA-Based Prediction of the Prognosis and Immunotherapy Response in Papillary Renal Cell Carcinoma

**DOI:** 10.3390/ijms24021464

**Published:** 2023-01-11

**Authors:** Yipeng Pang, Yushi Wang, Xinyu Zhou, Zhu Ni, Wenjing Chen, Yi Liu, Wenlong Du

**Affiliations:** 1 Department of Bioinformatics, School of Life Sciences, Xuzhou Medical University, Xuzhou 221004, China; 2 Department of Biophysics, School of Life Sciences, Xuzhou Medical University, Xuzhou 221004, China

**Keywords:** papillary renal cell carcinoma, prognostic markers, cuproptosis, long non-coding RNA (lncRNA), immune microenvironment, drug sensitivity

## Abstract

Cuproptosis, a new cell death pattern, is promising as an intervention target to treat tumors. Abnormal long non-coding RNA (lncRNA) expression is closely associated with the occurrence and development of papillary renal cell carcinoma (pRCC). However, cuproptosis-related lncRNAs (CRLs) remain largely unknown as prognostic markers for pRCC. We aimed to forecast the prognosis of pRCC patients by constructing models according to CRLs and to examine the correlation between the signatures and the inflammatory microenvironment. From the Cancer Genome Atlas (TCGA), RNA sequencing, genomic mutations and clinical data of TCGA-KIRP (Kidney renal papillary cell carcinoma) were analyzed. Randomly selected pRCC patients were allotted to the training and testing sets. To determine the independent prognostic impact of the training characteristic, the least absolute shrinkage and selection operator (LASSO) algorithm was utilized, together with univariate and multivariate Cox regression models. Further validation was performed in the testing and whole cohorts. External datasets were utilized to verify the prognostic value of CRLs as well. The CRLs prognostic features in pRCC were established based on the five CRLs (AC244033.2, LINC00886, AP000866.1, MRPS9-AS1 and CKMT2-AS1). The utility of CRLs was evaluated and validated in training, testing and all sets on the basis of the Kaplan–Meier (KM) survival analysis. The risk score could be a robust prognostic factor to forecast clinical outcomes for pRCC patients by the LASSO algorithm and univariate and multivariate Cox regression. Analysis of Gene Ontology (GO) and Kyoto Encyclopedia of Genes and Genomes (KEGG) data demonstrated that differentially expressed genes (DEGs) are primarily important for immune responses and the PI3K-Akt pathway. Arachidonic acid metabolism was enriched in the high-risk set by Gene Set Enrichment Analysis (GSEA). In addition, Tumor Immune Dysfunction and Exclusion (TIDE) analysis suggested that there was a high risk of immune escape in the high-risk cohort. The immune functions of the low- and high-risk sets differed significantly based on immune microenvironment analysis. Finally, four drugs were screened with a higher sensitivity to the high-risk set. Taken together, a novel model according to five CRLs was set up to forecast the prognosis of pRCC patients, which provides a potential strategy to treat pRCC by a combination of cuproptosis and immunotherapy.

## 1. Introduction

Up to 80% of kidney tumors are renal cell carcinomas (RCCs); The most common type of RCC is clear cell renal cell carcinoma (ccRCC), occupying approximately 75% of all cases, followed by papillary renal cell carcinoma (pRCC) [1,2]. It has been reported that for 70% of patients with localized pRCC, the overall survival was 5 years, while with advanced pRCC, no treatment options were available [3,4,5]. About 75% of RCCs belong to ccRCCs, which have been studied most in terms of biomarkers and target therapies, resulting in few solutions for treating patients with pRCC [6]. What is worse, pRCC is a highly heterogeneous disease with general chemo- and radio-resistance [7]. Thus, accurate biomarkers and effective treatments are needed for pRCC.

Copper ion is an extremely indispensable metal trace element and cofactor for organisms, which is involved in many biological processes. The amount of copper in the body is tightly regulated and toxic when it exceeds a threshold [8]. It has been reported in human cells that the control of cell death by copper which relies on mitochondrial respiration is unlike other known cell deaths, for instance, apoptosis, pyroptosis, necroptosis and ferroptosis [9]. Tsvetkov et al. found that copper-dependent cell death is due to the copper ions binding to the lipoylated elements of the tricarboxylic acid cycle (TCA), which causes the lipoylated proteins to accumulate and proteins linked to iron–sulfur clusters to be deficient, ultimately leading to a stress response and cell death [9]. Thus, researchers named this uncharacterized cell death as “cuproptosis”. A link between diseases and copper ions has also been reported. Copper ion levels are significantly elevated in several cancers [10,11,12]. Accumulation of copper ions has been shown to enhance cell proliferation, growth, angiogenesis and metabolism [13]. Copper ion imbalance is an critical element in the development and occurrence of cancer [14]. A novel approach to treat cancer based on copper toxicity has been mentioned by Kahlson et al. [15]. Therefore, this new cell death pattern is promising as an intervention target to treat tumors.

Long non-coding RNAs (LncRNAs) are molecules longer than 200 nucleotides, which play a key role in many diseases [16,17,18]. Tumors with abnormal expression of lncRNA are more likely to occur and develop, including RCC [19,20,21]. For instance, it has been reported that lncRNA ENTPD3-AS1 upregulation could suppress RCC via miR-155/HIF-1α pathway [19]. The tumor progression of RCC could be promoted by lncRNA SNHG12 via the upregulation of CDCA3 [20]. In addition, lncRNA RCAT1 could promote the progression of tumors and metastasis through miR-214-5p/E2F2 in RCC [21]. CRLs, including FOXD2-AS1, SUCLG2-AS1, LINC00271, NUP153-AS1 and LINC02154 have been used to predict prognosis and subtype in ccRCC [22]. It has also been reported that CRLs could be predictors to forecast prognosis in many other cancers, such as hepatocellular carcinoma, lung adenocarcinoma, colon adenocarcinoma and gastric cancer [23,24,25,26]. Nevertheless, the relationship between the dysregulation of CRLs and cancer development is not well understood in pRCC. Until now, there was no reference reporting the regulatory correlation between CRLs and pRCC. Exploring their relationship could be of great benefit in identifying potential tumor markers as therapeutic targets in pRCC.

In this study, we aimed to develop a prognostic model for an assessment and improvement of the prognosis of pRCC using CRLs, as well as to investigate the differences in the tumor microenvironment (TME), immunotherapy and drug response in pRCC. Therefore, a novel prognostic model was constructed according to five prognostic CRLs (AC244033.2, LINC00886, AP000866.1, MRPS9-AS1 and CKMT2-AS1), which has outstanding diagnostic value for patients with pRCC and is practical to guide clinicians to personalized immunotherapy and medication for pRCC patients according to individual differences.

## 2. Results

### 2.1. The Expression of Prognosis-Related lncRNAs Associated with the Coexpression of Cuproptosis Genes in pRCC

**Figure 1:**
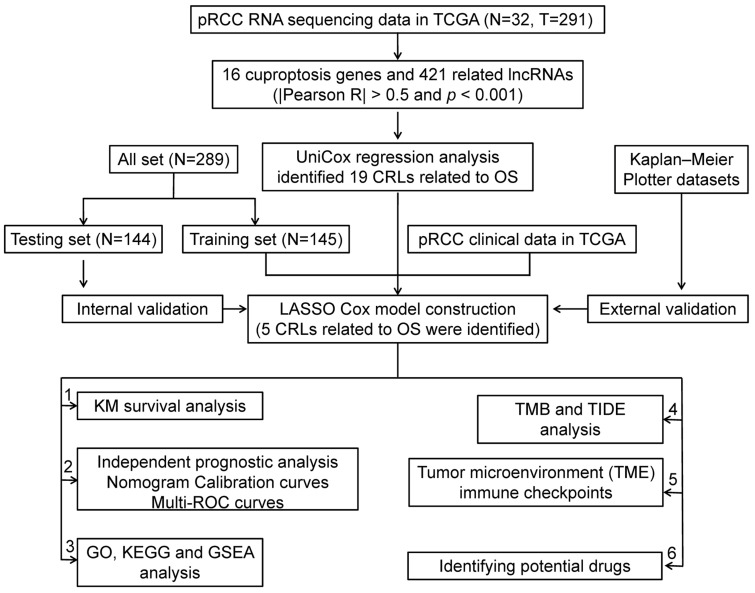
Flow chart of this study.

**Figure 2:**
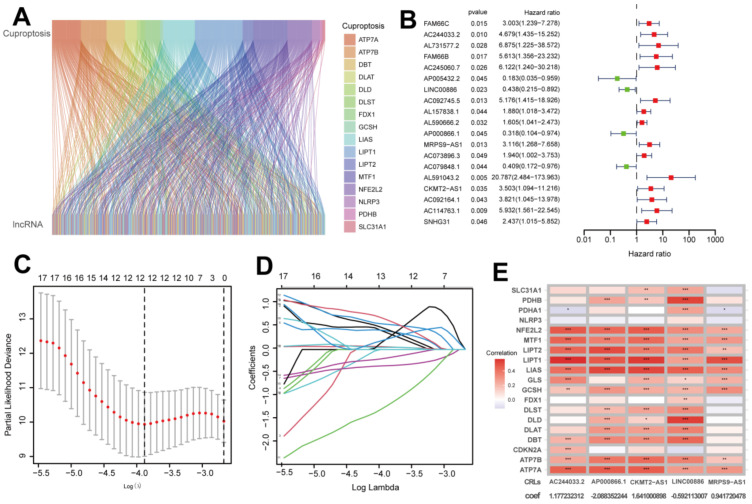
Identifying the prognostic features of papillary renal cell carcinoma (pRCC) linked to cuproptosis-related lncRNAs (CRLs). (**A**) The sankey relation between cuproptosis genes and CRLs. (**B**) Forest map showing prognostic genes for CRLs. (**C**) Least absolute shrinkage and selection operator (LASSO) coefficients of CRLs. (**D**) Cross-validation of CRLs in the LASSO regression. (**E**) Multivariate Cox regression and correlation for cuproptosis genes and CRLs. *, *p* < 0.05; **, *p* < 0.01; ***, *p* < 0.001.

A flowchart of the study is shown in Figure 1. By comparing 291 pRCC tumors and 32 normal tissues, we identified 19 cuproptosis genes in the expression matrix (Supplementary Table S1). A total of 421 CRLs were obtained (Supplementary Table S2) based on Pearson analysis (|Pearson R| > 0.5 and *p* < 0.001). A total of 289 pRCC patients were randomly allotted to the training set (*n* = 145) and the testing set (*n* =144). The clinical features of the two sets of patients are presented in Table 1 showing that no clinical traits differed between the training and testing sets. CRLs were correlated with cuproptosis-related genes (Figure 2A). Then, 19 CRLs were derived from univariate and Lasso Cox regression analyses (Figure 2B–D). A total of 15 of the 19 CRLs with a hazard ratio (HR) > 1 were found to be bad prognostic predictors while the other 4, AP005432.2, LINC00886, AP000866.1 and AC079848.1, may be protective indicators (Figure 2B). The TCGA-KIRP cohort showed five candidate CRLs related to OS based on the multivariate Cox regression method (Figure 2E). Finally, the risk model was constructed using the five key CRLs (Supplementary Table S3), which included AC244033.2, LINC00886, AP000866.1, MRPS9-AS1 and CKMT2-AS1. Risk score = (1.177232312 × Expression_AC244033.2_) + (−0.592113007 × Expression_LINC00886_) + (−2.088352244 × Expression_AP000866.1_) + (0.941720478 × Expression_MRPS9-AS1_) + (1.641000898 × Expression_CKMT2-AS1_). The relationships between the five CRLs and cuproptosis genes are demonstrated in Figure 2E.

### 2.2. Predicting the Prognosis of pRCC Patients with the CRLs Predictive Signature

**Figure 3:**
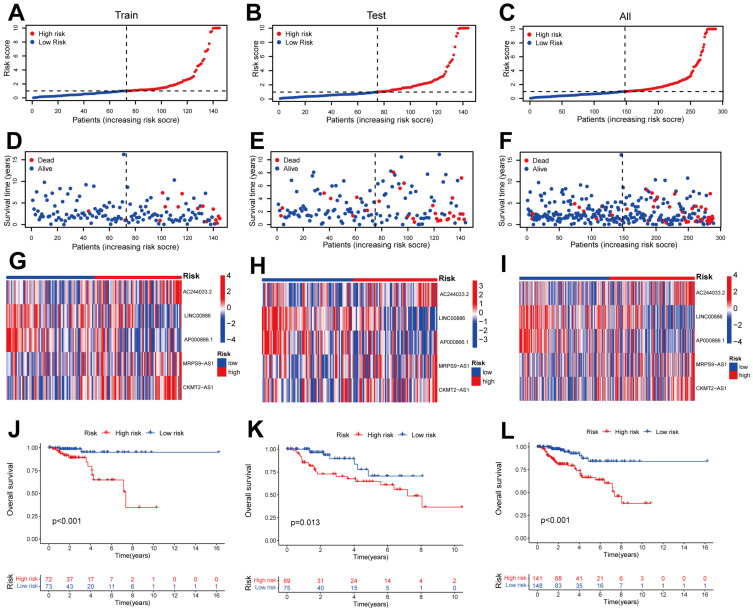
Evaluating and validating for the prognostic value of CRLs in testing, training and all sets. (**A**–**C**) Patient distribution with increasing risk scores. (**D**–**F**) Survival time of patients and risk scores. (**G**–**I**) Heatmaps of 5 CRLs. (**J**–**L**) Kaplan–Meier (KM) survival analysis of overall survival (OS) of pRCC patients between high- and low-risk sets.

**Figure 4:**
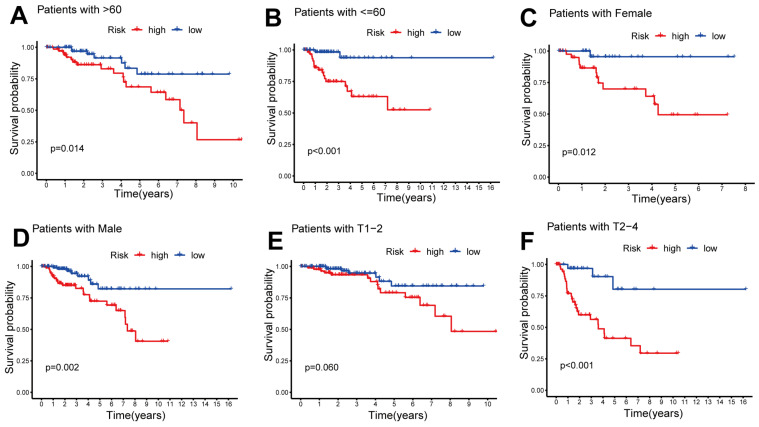
Kaplan–Meier (KM) survival analysis of low- and high-risk patients with different clinical covariates. Age (**A**,**B**), gender (**C**,**D**) and T stage (**E**,**F**).

Training patients were assigned into high- and low-risk sets for survival analysis according to their median risk scores. The results demonstrated that low-risk patients had significantly better OS than those in the high-risk set (*p* < 0.001; Figure 3J). With increasing risk scores, patients with pRCC had an increased mortality rate (Figure 3A,D). The same phenomenon was also observed in the testing and all sets (Figure 3B,C,E,F,K,L). AC244033.2, CKMT2-AS1 and MRPS9-AS1 expressed more in the high-risk set than in the low-risk set for training, testing and whole sets (Figure 3G–I), suggesting that these CRLs could be poor prognostic predictors. On the contrary, the expression of CRLs, LINC00886 and AP000866.1, was lower in the high-risk set than that in the low-risk set, indicating that these CRLs may be protective indicators (Figure 3G–I). Meanwhile, the survival probability and clinical features of pRCC patients were compared according to the age, gender and T stage. The results demonstrated that for all clinical variables except stage T1-2, high-risk patients had a shorter OS than low-risk patients, regardless of their clinical variables. (Figure 4). One possible explanation is the small number of T1-2 patients due to the bad prognosis of advanced pRCC. Based on our results, our model could be applied to different clinical variables.

### 2.3. The Risk Score Could Be a Robust Prognostic Factor to Predict Clinical Outcomes for pRCC Patients

**Figure 5:**
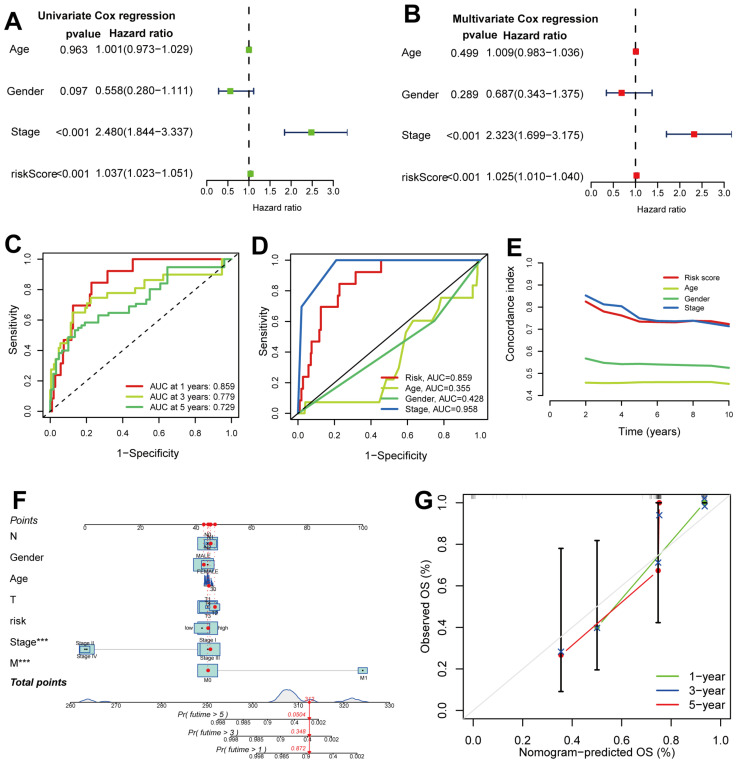
Clinical predictability and independent prognostic ability of five CRLs for patients with pRCC. (**A**) Clinical variables and signature CRLs in a univariate Cox regression. (**B**) A multivariate Cox regression analysis of clinical variables and signature CRLs. (**C**) Prediction 1-, 3- and 5-OS of pRCC patients in the entire set. (**D**) Comparison of predictive risk model and clinicopathological features. (**E**) C-index ROC curve of the risk model. (**F**) A nomogram showing risk and clinicopathological features for predicting 1-, 3- and 5-OS in pRCC patients. ***, *p* < 0.001. (**G**) Calibration curves indicating the accuracy of risk model to predict 1-, 3- and 5-OS of pRCC patients.

On the basis of univariate and multivariate Cox regression, the risk score was shown to be an independent prognostic factor for pRCC patients (Figure 5A,B). The area under the curve (AUC) for 1-, 3- and 5-year receiver operating characteristic curves (ROCs) was 0.859, 0.779 and 0.729, respectively (Figure 5C). AUC for the risk score was 0.859 (Figure 5D), suggesting better predictive power than other clinical features other than the stage. Consistent with the former results, ROC curves showed that the 10-year C-index scored higher in the risk model than other clinical variables (Figure 5E), demonstrating that the risk score may be a robust prognostic factor to forecast especially long-time clinical outcome for pRCC patients. Furthermore, the probability of 1-, 3- and 5-year overall survival (OS) for the selected patient was 0.872, 0.348 and 0.0504, respectively, on the basis of the clinical variables and risk scores included in the nomogram (Figure 5F). The nomogram predictions were validated by the calibration plots showing that clinical outcomes and predictive survival were in good agreement (Figure 5G). Taken together, these results suggest that the risk score could be a robust prognostic element in forecasting clinical outcomes for pRCC patients.

### 2.4. Gene Ontology (GO), Kyoto Encyclopedia of Genes and Genomes (KEGG) and Gene Set Enrichment Analysis (GSEA) for the High- and Low-Risk Sets

**Figure 6:**
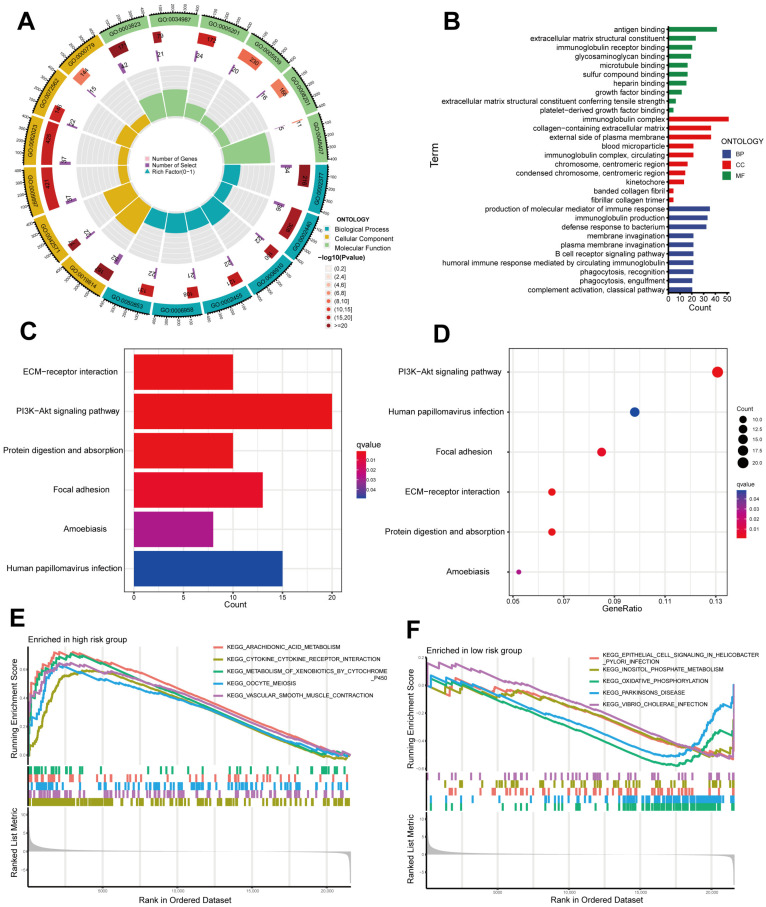
Gene Ontology (GO), Kyoto Encyclopedia of Genes and Genomes (KEGG) and Gene Set Enrichment Analysis (GSEA) between high- and low-risk sets. (**A**,**B**) GO analysis revealed the diversity of molecular biological processes (BPs), cellular components (CCs) and molecular functions (MFs). (**C**,**D**) Significantly enriched pathways were identified by KEGG pathway analysis. (**E**,**F**) GSEA demonstrating the top five enriched pathways in high- and low-risk sets.

First, the differentially expressed genes (DEGs) based on the average expression of samples from high- and low-risk sets were determined (Padjust < 0.05, |log_2_ (fold change)| ≥ 1, Supplementary Table S4). Then, to explore the DEGs of biological properties, we used the R package “clusterprofiler” to conduct GO and KEGG enrichment analysis. As demonstrated by biological process (BP) terminology, DEGs contribute greatly to the “production of molecular mediator of immune response”, “immunoglobulin production” and “defense response to bacterium”. In the field of cellular components (CC), “immunoglobulin complex”, “external side of plasma membrane” and “collagen-containing extracellular matrix” were significantly abundant. DEGs were enriched for molecular functions (MFs) related to “antigen binding”, “extracellular matrix structural constituent” and “immunoglobulin receptor binding” (Figure 6A,B, Supplementary Table S5). These results indicated that DEGs mainly contributed to the development of immune responses. In addition, KEGG analysis indicated that DEGs of low- and high-sets were enriched in the “PI3K-Akt signaling pathway”, “Human papillomavirus infection” and “Focal adhesion” (Figure 6C,D). Furthermore, in the high-risk set, “arachidonic acid metabolism” and “metabolism of xenobiotic by cytochrome p450” were activated (Figure 6E). There was a significant enrichment of “oxidative phosphorylation” and “parkinsons disease” for the low-risk patients (Figure 6F).

### 2.5. The Mutational Landscape for pRCC and Tumor Mutational Burden (TMB) Together with Tumor Immune Dysfunction and Exclusion (TIDE) Analysis

**Figure 7:**
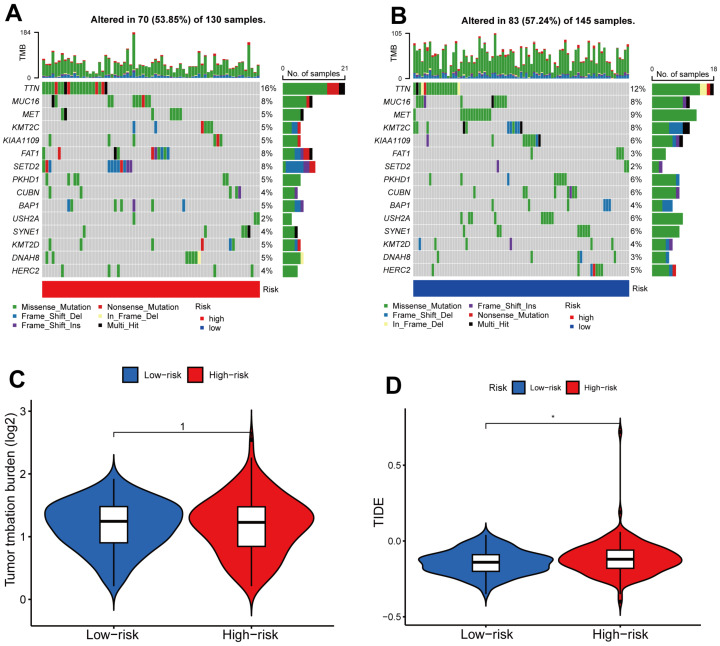
Tumor Mutational Burden (TMB) and Tumor Immune Dysfunction and Exclusion (TIDE) analysis. (**A**) The waterfall plot showed the TMB of 15 genes in high-risk set. (**B**) The waterfall plot showed the TMB for 15 genes in the low-risk set. (**C**) Analysis of the difference for TMB between low- and high-risk patients. (**D**) TIDE analysis for low- and high-risk sets. *, *p* < 0.05.

To analyze the changes of somatic mutations in high- and low-risk sets, we downloaded the somatic mutation data from the TCGA database. The 15 most highly mutated genes were TTN, MUC16, MET, KMT2C, KIAA1190, FAT1, SETD2, PKHD1, CUBN, BAP1, USH2A, SYNE1, KMT2D, DNAH8 and HERC2 (Figure 7A,B). TTN, FAT1 and SETD2 mutations were evidently more common in the high-risk set than in the low-risk population (Figure 7A,B). However, in an overall view, low- and high-risk sets did not exhibit significant differences in tumor mutational burden (Figure 7C). In addition, a higher TIDE score was observed in the high-risk set than in the low-risk set (Figure 7D). The results indicate that immunotherapy may have a better therapeutic effect on low-risk patients due to a lower potential for immune escape.

### 2.6. Tumor Immune Microenvironment Landscape of pRCC in High- and Low-Risk Patients

**Figure 8:**
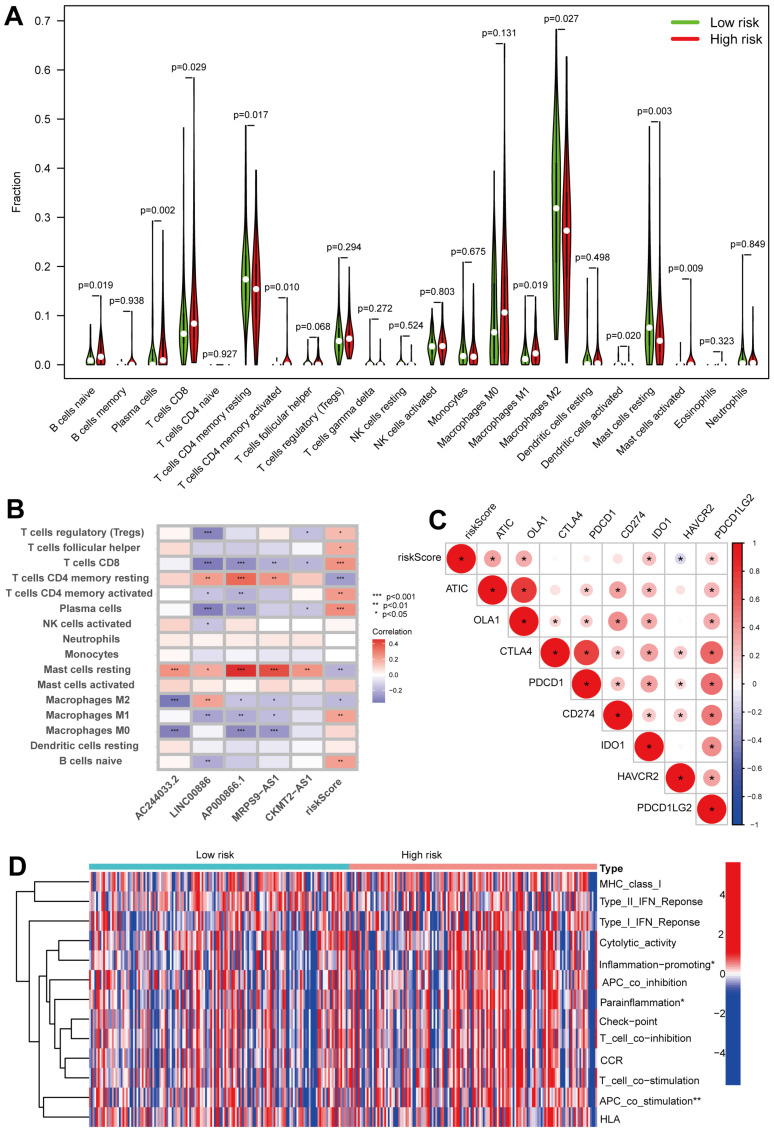
Analysis of the tumor microenvironment (TME) between the high- and low-risk patients. (**A**) Violin plot showing the fraction of 22 types of tumor-infiltrating immune cells for the low- and high-risk sets. (**B**) Correlation between infiltrating immune cells and 5 prognostic CRLs together with risk score. (**C**) An analysis of the correlation between immune checkpoints and risk scores. *, *p* < 0.05. (**D**) The heat map depicting an analysis of the immune function differences between the groups at low and high risk. *, *p* < 0.05; **, *p* < 0.01.

Due to the central role of the tumor immune microenvironment and the revolutionary effect immune checkpoint inhibitors play in treating tumors, we surveyed the relationship between the immune microenvironment and the risk score in all patients. According to the CIBERSORT algorithm, we calculated the percentage of tumor-infiltrating immune cells in high- and low-risk sets of pRCC (Supplementary Table S6). As demonstrated in Figure 8A, patients in the high-risk set had significantly lower proportions of tumor-infiltrating CD4 memory resting T cells, M2 macrophages and resting mast cells. High-risk patients, however, had significantly higher proportions of tumor-infiltrating native B cells, plasma cells, CD8 T cells, CD4 memory-activated T cells, M1 macrophages and activated mast cells. Furthermore, a correlation was shown in Figure 8B between the risk score and the abundance of tumor-infiltrating immune cells. According to the heat map, the risk score was positively associated with the abundance of regulatory (Tregs) T cells, follicular helper T cells, CD8 T cells, CD4 memory-activated T cells, plasma cells, M1 macrophages and native B cells, and negatively associated with CD4 memory-resting T cells, resting mast cells and M2 macrophages (Figure 8B). In addition, five prognostic CRLs were correlated with tumor-infiltrating immune cells as well (Figure 8B). We noted that a significant association was found between these five CRLs and most immune cells (Figure 8B). In particular, resting mast cells were positively correlated with all of the five CRLs (Figure 8B). Moreover, analyses were performed on the relationship between immune checkpoints and risk scores. Interestingly, among the immune checkpoints, half were positively associated with the risk score, and only one was negatively associated (Figure 8C), which may explain that patients had a poorer OS with a higher risk score. Finally, the differences in immune-related functions in the high- and low-risk sets were surveyed using GSVA package ssGSEA. As depicted in Figure 8D, “inflammation-promoting”, “parainflammation” and “APC_co_stimulation” were remarkably more enriched in the high-risk cohort.

### 2.7. Screening Potential Drugs for pRCC

**Figure 9:**
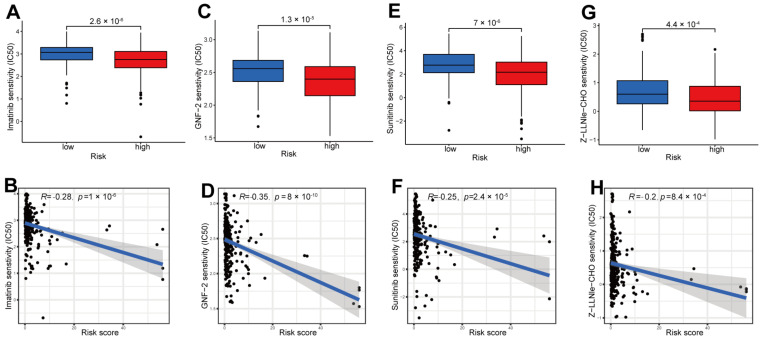
Identifying potential drugs for the treatment of pRCC. IC50 differences between high- and low-risk sets for four drugs: imatinib (**A**,**B**), GNF-2 (**C**,**D**), sunitinib (**E**,**F**) and Z-LLNIe-CHO (**G**,**H**).

To screen the potentially effective anti-tumor drugs, we utilized the pRRophetic packages. Four drugs including imatinib (Figure 9A,B), GNF-2 (Figure 9C,D), sunitinib (Figure 9E,F) and Z-LLNIe-CHO (Figure 9G,H) were screened based on their higher sensitivity/lower IC50 to the high-risk set, demonstrating that high-risk patients with pRCC are more likely to benefit from these four drugs.

### 2.8. External Datasets Validation of CRLs as Potential Biomarkers of pRCC

**Figure 10:**
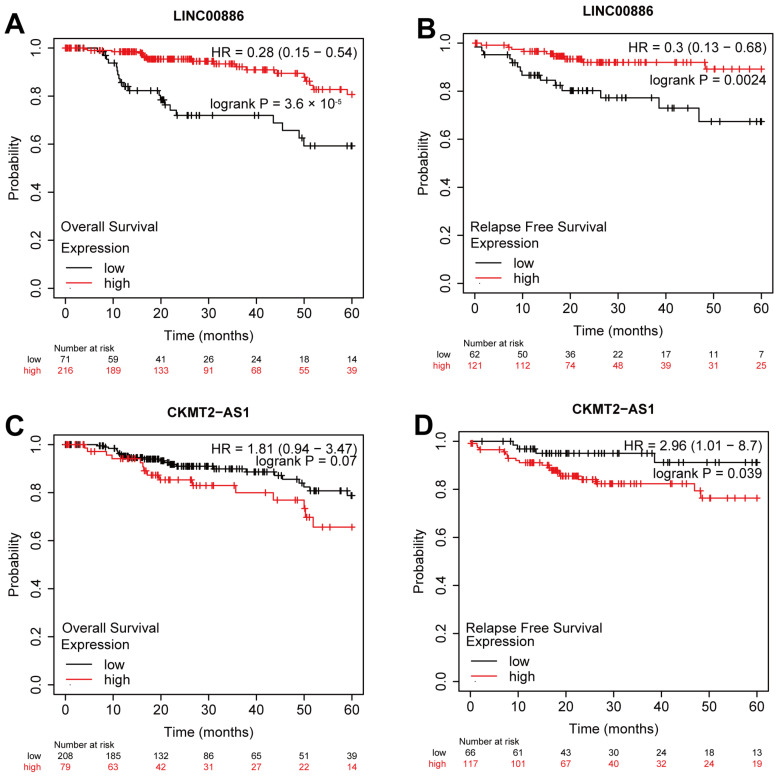
External datasets validation of CRLs as possible biomarkers. Overall survival (OS) and relapse free survival (RFS) analysis of LINC00886 (**A**,**B**) and CKMT2-AS1 (**C**,**D**) from the Kaplan–Meier Plotter datasets.

Based on the external KM Plotter database, LINC00886 and CKMT2-AS1 were analyzed for prognostic value, while the remaining three CRLs were absent in the database. The results indicated that an important protective prognostic factor, LINC00886, had a strong correlation with OS (HR = 0.28 (0.15–0.54), *p* = 3.6 × 10^−5^) (Figure 10A) and RFS (HR = 0.3 (0.13–0.68), *p* = 0.0024) (Figure 10B). On the contrary, a biomarker of poor prognosis, CKMT2-AS1, was significantly correlated with RFS (HR = 2.96 (1.01–8.7), *p* = 0.039) (Figure 10D) but not OS (Figure 10C). Our data were in agreement with the results of the survival analysis from external database.

## 3. Discussion

In this study, five prognostic CRLs, including AC244033.2, LINC00886, AP000866.1, MRPS9-AS1 and CKMT2-AS1 were identified by bioinformatic analysis, which had high accuracy in forecasting overall survival (OS). It was found in our study that these five prognostic CRLs were closely related to TME, immunotherapy and drug response in pRCC.

Although some targeted agents have been utilized clinically in patients with highly differentiated RCC, their efficacy in pRCC has not been proven [27,28]. Because pRCCs are rare, genetic testing and randomized controlled trials are usually not included or only account for a small percentage of RCC cases [29]. Moreover, the relative findings concerning ccRCC do not apply to pRCC owing to the difference between the two subtypes of RCC [30]. Therefore, to find new biomarkers for targeted therapy, it is essential to research the molecular mechanisms of these diseases.

Recent studies have shown that mitochondrial lipid acylated protein accumulation and the loss of Fe–S cluster proteins caused by intracellular copper accumulation could eventually result in proteotoxic stress-induced cell death, known as cuproptosis [9]. Studies of copper toxicity can be conducted using copper ionophores that are required for intracellular copper accumulation [31]. Due to the fact that cuproptosis induces cancer cells in a preferential manner in comparison to normal cells, copper ionophores have great potential in cancer therapy [11,32,33,34]. Thus, studies related to cuproptosis are urgent for a deeper understanding of the cuproptosis application on cancer therapy.

It has been reported that lncRNAs are critical to the pRCC progression in several studies. Chen et al. found that the up-regulation of lncRNA RP11-63A11.1 could attenuate cell survival and induce apoptosis in pRCC [35]. In another study, the utility of immune-related lncRNAs (IR-lncRNAs) to forecast prognosis and tumor progression in pRCC patients was explored by Liu et al. [36]. However, CRLs in pRCC have never been investigated. Here we constructed risk model according to CRLs to forecast OS of patients with pRCC. A total of 19 CRLs correlated with prognosis were acquired by analysis. Then, five CRLs including AC244033.2, LINC00886, AP000866.1, MRPS9-AS1 and CKMT2-AS1, were screened according to LASSO, univariate and multivariate Cox regression analysis. We constructed the characteristics of CRLs using the aforementioned five CRLs to forecast the prognosis of pRCC patients. Among these CRLs, esophageal squamous cell carcinomas were reported to be suppressed by lncRNA LINC00886 through the SIRT7/ELF3/miR-144 pathway [37]. It also could serve as a tumor suppressor to repress the malignant progression of laryngeal carcinoma [38]. These results are consistent with our data that lncRNA LINC00886 may be a protective indicator in pRCC due to its low expression in high-risk set (Figure 3G–I), which was also confirmed by the validation of external datasets (Figure 10A,B). On the contrary, lncRNA LINC00886 could be a risk factor rather than a protective indicator for glioma patients [39]. The high-risk patients with colon cancer showed an elevated expression level of MRPS9-AS1 [40], which is consistent with our results (Figure 3G–I), indicating that lncRNA MRPS9-AS1 is linked to a poor prognosis of pRCC. In another study, lncRNA CKMT2-AS1 depressed the growth of colorectal cancer cells by targeting the AKT/mTOR pathway [41], which contradicts our data that lncRNA CKMT2-AS1 could be a poor indicator for pRCC rather than a protective factor (Figure 3G–I). CKMT2-AS1 as a bad indicator was further verified by external KM survival analysis (Figure 10D). The remaining two CRLs (AC244033.2 and AP000866.1) are uncharacterized in other cancers. These CRLs could help us understand pRCC better and identify new possible targets for therapeutic intervention.

Here, we divided the TCGA-KIRP cohort into training and testing sets randomly. Using the median risk score from the training set, the training and testing sets were classified as high- and low-risk, respectively. The clinical statistical analysis of the high- and low- risk sets suggested that there was no difference between the two sets on clinical features (Table 1). Then, high-risk patients had a poor prognosis on the basis of survival curve results (Figure 3). Subsequently, in different clinical variables, survival curve analysis showed a lower OS in the patients with high risk than in those with low risk, except for stage T1-2. (Figure 4). One possible explanation is the small number of T1-2 patients due to the bad prognosis of advanced pRCC. The risk score could be a robust prognostic factor to forecast clinical outcomes for pRCC patients according to univariate, multivariate Cox regression, ROC, C-index and calibration curves combined with nomogram (Figure 5). On the basis of GO analyses, DEGs were mainly involved in immune system development (Figure 5A,B). KEGG analysis indicated that the “PI3K-Akt signaling pathway” and “Human papillomavirus infection” were enriched in CRLs (Figure 6C,D). The PI3K-Akt pathway can initiate downstream signaling molecules that contribute to the invasion and metastasis of tumor cells [42]. “Arachidonic acid metabolism” and “metabolism of xenobiotic by cytochrome p450” were activated in the high-risk set by GSEA analysis (Figure 6E). Several cancers and inflammatory diseases are associated with the arachidonic acid pathway [43], which could be a novel preventive and therapeutic target for pRCC. TIDE analysis demonstrated that high-risk patients had a high likelihood of escaping immunity (Figure 7D), indicating that the high-risk patients may obtain a poor therapeutic effect from immunotherapy and react more limitedly to immune checkpoint inhibitors (ICI) therapy. In addition, there was a higher expression of immune checkpoints, including ATIC, OLA1, IDO1 and PDCD1LG2 (PD-L2), in the high-risk set than the low-risk set (Figure 8C). However, whether the inhibitors of these checkpoints could act as promising antitumor agents for pRCC needs further research. It has been reported that PD-L2 in ccRCC was positively related with progression-free survival and cancer-specific survival, but not in pRCC [44]. Thus, PD-L2 targeted immunotherapy may be useful for ccRCC but not pRCC. Finally, four potential drugs, including imatinib, GNF-2, sunitinib and Z-LLNIe-CHO were screened, which had a lower IC50 in high-risk set (Figure 9). Among these agents, sunitinib was reported to develop into targeted agents for RCC [45]. The remaining three agents have not been described in terms of pRCC. Our results suggested that imatinib, GNF-2 and Z-LLNIe-CHO may be potential agents for pRCC and provided a research strategy for improving the clinical efficacy of pRCC.

The study we conducted had some limitations. Since these findings were derived from the TCGA database and further experiments are needed to verify their accuracy. In addition, we could not obtain proper lncRNA and clinical information of pRCC from the GEO database. Thus, two of these lncRNAs were validated using the external Kaplan-Meier Plotter database including GEO, EGA and TCGA.

## 4. Materials and Methods

### 4.1. Data Acquisition

Clinical and RNA sequencing data of pRCC were downloaded from TCGA database (https://portal.gdc.cancer.gov/repository (accessed on 15 October 2022)), which included 291 pRCC tumors and 32 normal tissues. Language Perl (version Strawberry—perl-5.30.0; https://www.perl.org (accessed on 15 October 2022)) was employed to extract RNA-seq data from the standardized FPKM format [46]. Pathology information was extracted from clinical data using Pearl.

### 4.2. Profiles of Differential Expressions in CRLs Screened and Characterized

The cuproptosis-related expression matrix was obtained using R packages “BiocManager” and “limma” [47]. CRLs expression matrix was also obtained with packages “BiocManager” and “limma” [47] (|Pearson R| > 0.5 and *p* < 0.001). Then, the sankey relationship between cuproptosis genes and CRLs was plotted with packages “ggplot2” and “ggalluvial” [48].

### 4.3. Prognostic Risk Modeling and Validation

In a 1:1 ratio, pRCC patients were randomly allotted to the training and testing sets with the package “caret”. The train set was utilized to build the CRLs prognostic model. Model validation was based on the testing and entire sets. Prognostic CRLs were acquired via univariate Cox regression (*p* < 0.05). A total of 5 CRLs were obtained by the LASSO algorithm and univariate and multivariate Cox regression analysis. The related maps were drawn with packages “survival”, “glmnet”, “survminer” and “timeROC” [49,50]. Finally, a risk score was calculated for each patient according to the following formula:

risk score = Σi = lnCoef (i) × Expr (i).

Regression coefficient and expression of lncRNA are represented by Coef (i) and Expr (i), respectively. High- and low-risk sets were divided in training, testing, and whole sets, respectively, according to the median risk score for the training set. ROC curves together with C-index were utilized to measure the accuracy of the model with packages “survminer”, “timeROC”, “survival”, “rms” and “pec” [51]. To evaluate model accuracy in forecasting prognosis, nomograms and calibration were plotted with packages “survival”, “regplot”, and “rms”.

### 4.4. GO, KEGG and GSEA Analysis

GO analysis was performed with packages “colorspace”, “stringi”, “ggplot2”, “circlize”, “RColorBrewer” and “ggpubr” to demonstrate the DEGs of high- and low-risk sets [52]. KEGG analysis was carried out with packages “BiocManager”, “org.Hs.eg.db”, “DOSE”, “clusterProfiler”, “enrichplot” and “ComplexHeatmap” to reveal the pathways associated with high- and low-risk sets [53,54,55]. GSEA analysis was performed with packages “org.Hs.eg.db”, “limma”, “clusterProfiler” and “enrichplot” to further identify the enriched pathways in different sets.

### 4.5. Tumor Mutation Burden (TMB) and Immune Dysfunction and Exclusion (TIDE) Analysis

The package “maftools” was utilized to analyze the differences in TMB between the high- and low-risk sets. Scoring file for TIDE was downloaded from the website (http://tide.dfci.harvard.edu, (accessed on 15 October 2022)) [56].

### 4.6. Tumor Microenvironment (TME) Analysis

For low- and high-risk sets, violin plots were created with the packages “limma” and “vioplot”, showing the percentage of 22 types of tumor-infiltrating immune cells. We utilized packages “limma”, “reshape2”, “tidyverse”, “ggplot2”, “ggpubr” and “ggExtra” to survey the correlation among risk score, infiltrating immune cells and immune checkpoints. The heat map exhibited the difference of immune-related functions for the low- and high-risk sets, which was plotted with packages “limma”, “GSVA”, “GSEABase”, “pheatmap” and “reshape2”. Furthermore, the IC50 values of drugs available for treating pRCC in high-risk and low-risk sets were predicted by pRRophetic.

### 4.7. External Dataset Validation

CRLs were tested for prognostic value using a KM plotter database (https://kmplot.com/analysis/ (accessed on 15 October 2022)). Auto selecting best cutoff was utilized to split patients.

## 5. Conclusions

Overall, a novel prognostic model based on five CRLs was developed for pRCC patients. The features were correlated with the immune microenvironment, immune checkpoints and immunotherapy. The findings will contribute to a better understanding of the underlying mechanisms of pRCC emergence and progression, which provide a potential strategy to treat pRCC by the combination of cuproptosis and immunotherapy.

## Figures and Tables

**Table 1:** The clinical features of pRCC patients who were trained and tested.

Covariates	Total (*n* = 289)	Testing Set (*n* = 144)	Training Set (*n* = 145)	*p* Value
Age				
<=65	179 (61.94%)	90 (62.5%)	89 (61.38%)	0.9394
>65	108 (37.37%)	53 (36.81%)	55 (37.93%)	-
unknown	2 (0.69%)	1 (0.69%)	1 (0.69%)	-
Gender				
Female	76 (26.3%)	43 (29.86%)	33 (22.76%)	0.2158
Male	213 (73.7%)	101 (70.14%)	112 (77.24%)	-
Stage				
Stage-I	171 (59.17%)	95 (65.97%)	76 (52.41%)	0.1082
Stage-II	21 (7.27%)	12 (8.33%)	9 (6.21%)	-
Stage-III	52 (17.99%)	19 (13.19%)	33 (22.76%)	-
Stage-IV	15 (5.19%)	8 (5.56%)	7 (4.83%)	-
unknown	30 (10.38%)	10 (6.94%)	20 (13.79%)	-
T				
T1	192 (66.44%)	101 (70.14%)	91 (62.76%)	0.3062
T2	33 (11.42%)	17 (11.81%)	16 (11.03%)	-
T3	60 (20.76%)	26 (18.06%)	34 (23.45%)	-
T4	2 (0.69%)	0 (0%)	2 (1.38%)	-
unknown	2 (0.69%)	0 (0%)	2 (1.38%)	-
M				
M0	95 (32.87%)	44 (30.56%)	51 (35.17%)	0.4129
M1	9 (3.11%)	6 (4.17%)	3 (2.07%)	-
unknown	185 (64.01%)	94 (65.28%)	91 (62.76%)	-
N				
N0	50 (17.3%)	27 (18.75%)	23 (15.86%)	0.9874
N1	24 (8.3%)	13 (9.03%)	11 (7.59%)	-
N2	4 (1.38%)	2 (1.39%)	2 (1.38%)	-
unknown	211 (73.01%)	102 (70.83%)	109 (75.17%)	-

## Data Availability

Not applicable.

## Supplementary Materials

The following supporting information can be downloaded at: https://www.mdpi.com/article/10.3390/ijms24021464/s1.
